# Room-Temperature Nitric Oxide Gas Sensors Based on NiO/SnO_2_ Heterostructures

**DOI:** 10.3390/s23208583

**Published:** 2023-10-19

**Authors:** Emmanouil Gagaoudakis, Apostolos Tsakirakis, Marilena Moschogiannaki, Angeliki Sfakianou, Vassilios Binas

**Affiliations:** 1 Institute of Electronic Structure and Laser, Foundation for Research and Technology-Hellas (FORTH-IESL), 700 13 Heraklion, Greece; kanwotithelw776@gmail.com (A.T.); marilenamosho@hotmail.com (M.M.); asfakianou@physics.uoc.gr (A.S.); binasbill@iesl.forth.gr (V.B.); 2 Department of Materials Science and Technology, University of Crete, 700 13 Herakleion, Greece; 3 Department of Physics, University of Crete, 700 13 Herakleion, Greece; 4 Department of Chemistry, Aristotle University of Thessaloniki, 541 24 Thessaloniki, Greece

**Keywords:** NiO/SnO_2_ heterostructure, polyol process, thermal annealing, nitric oxide detection, room temperature

## Abstract

Nitric oxide (NO) is a very well-known indoor pollutant, and high concentrations of it in the atmosphere lead to acid rain. Thus, there is great demand for NO sensors that have the ability to work at room temperature. In this work, NiO/SnO_2_ heterostructures have been prepared via the polyol process and were tested against different concentrations of NO gas at room temperature. The structural and morphological characteristics of the heterostructures were examined using X-ray diffraction and scanning electron microscopy, respectively, while the ratio of NiO to SnO_2_ was determined through the use of energy-dispersive spectrometry. The effects of both pH and thermal annealing on the morphological, structural and gas-sensing properties of the heterostructure were investigated. It was found that the morphology of the heterostructures consisted of rod-like particles with different sizes, depending on the temperature of thermal annealing. Moreover, NiO/SnO_2_ heterostructures synthesized with pH = 8 and annealed at 900 °C showed a response of 1.8% towards 2.5 ppm NO at room temperature. The effects of humidity as well as of stability on the gas sensing performance were also investigated.

## 1. Introduction

Nitric oxide (NO), a well-known dangerous environmental pollutant, is a product of fossil fuel combustion and is found in emissions. Thus, its detection is crucial in terms of monitoring air quality. It is responsible for a number of human diseases, such as irritation to the skin or eyes and respiratory malfunctions, such as asthma, etc. [1,2]. Specifically, NO acts as a biomarker for asthma detection because its production by airway cells is strongly related to diseased cells. It has been found that the NO concentration exhaled by breath increased from 33 ppb for a healthy individual to about 98 ppb for an asthma patient [3,4,5]. In addition, the threshold limit value (TLV) for NO is 25 ppm for an 8 h exposure, according to the Occupational Safety and Health Organization (OSHA) [6]. As a result, there is a high demand for NO gas sensors that can be prepared using cost-effective methods and work at room temperature (RT).

In order to meet the above-mentioned requirements, a great number of materials have been tested as sensing elements against NO gas and using various sensing techniques, such as looking for a change in electrical resistance (chemoresistive sensor) [7], optical sensors [8] or surface acoustic wave devices [9,10]. Among them, chemoresistive sensors are by far the most investigated due to the simplicity of the measurement as well as the great variety of materials that can be used in this technique; however, most of them require a high operating temperature [1,2,11,12,13,14] or UV irradiation [15,16,17] in order to detect NO gas concentrations below the TLV, leading to extra energy consumption. Thus, only a few works have been reported on NO detection at room temperature. Specifically, Chang et al. [18] used N-doped reduced GO to detect 1 ppm NO with a 1.7 response, while Gaussian et al. [19] succeeded in detecting 100 ppm NO with a response of 80.7% and a response time of 300 s. Finally, Kuchi et al. [20] grew a TiO_2_-rGO heterostructure in order to detect 2.75 ppm NO for a 7.1 response and a 440 s response time.

In this work, NiO/SnO_2_ heterostructures synthesized using a polyol process were tested as room temperature NO gas sensors at concentrations far below the TLV. SnO_2_ is a well-known n-type semiconductor that has been extensively studied as a gas sensor [21,22,23,24] in relation to numerous gases, such as NO_2_ [25], SO_2_ [26], H_2_ [27], CH_4_ [28], etc. Moreover, SnO_2_ was the first material that was used in commercial chemoresistive gas sensors due to its stability and reliability [24]. Recently, it has been used in combination with a p-type metal oxide semiconductor, forming a p-n heterostructure, in order to enhance gas sensing performance. More specifically, p-n heterostructures of CuO/SnO_2_ [29], Co_3_O_4_/SnO_2_ [30] and NiO/SnO_2_ [31,32] have successfully detected H_2_S, NH_3_, SO_2_ and formaldehyde, respectively. Apart from these, NiO/SnO_2_ heterostructures have also been tested against ethanol [33], triethylamine [34] and n-butanol [35]. Another issue that the gas sensors have to deal with is the lack of selectivity due to the interference of various gases [36]. Herein, the heterostructures were also tested against other gases, showing no response, indicating a selectivity toward NO.

Furthermore, NiO/SnO_2_ p-n heterostructures have been applied to a great number of applications, such as anode materials for lithium-ion batteries [37], energy storage devices [38], photocatalysis [39], UV photodetectors [40], etc.; moreover, they can also be synthesized using a variety of methods, including sputtering [31], hydrothermal reactions [32], and electrospinning [35], which leads to different morphologies. Herein, NiO/SnO_2_ heterostructures grown using a simple and cost-effective chemical method were deposited via the spin coating method on interdigitated electrodes (IDEs)/glass substrates. The NiO/SnO_2_ sensor successfully detected 1 ppm NO at room temperature, making it a possible NO gas sensing material.

## 2. Materials and Methods

### 2.1. Synthesis of NiO/SnO_2_ Heterostructure

NiCl_2_·6H_2_O (>98%, Sigma Aldrich, St. Louis, MI, USA) and SnCl_2_·2H_2_O (98%, Alfa Aesar, Haverhill, MA, USA) were used as Ni and Sn sources, respectively; ethylene glycol, C_2_H_6_O_2_ (≥99.5%, Merck, Rahway, NJ, USA) was used as a solvent, while ammonia NH_3_, (25% in water, Sigma Aldrich, St. Louis, MI, USA) was used in order to regulate the pH. All of the purchased reagents were of analytical grade and used without further purification. In a typical procedure, 0.475 g NiCl_2_·6H_2_O and 0.521 g SnCl_2_·2H_2_O were solved in 30 mL of ethylene glycol. The solution was stirred for 1 h, after which the pH was 0.3, and the solution a transparent green color. By adding ammonia, solutions with 2 different pH values, namely 6 and 8, were prepared, having the colors green and light blue, respectively, and they were semitransparent. After 1 h of stirring, the solutions were centrifuged for 15 min at 4000 rpm, and the precipitate was washed two times with ethanol and dried for 1 day at 70 °C. The obtained powder was thermally annealed at 300 °C, 600 °C and 900 °C for 2 h with a step of 2 °C/min.

### 2.2. Fabrication of NiO/SnO_2_ Sensor

The gas sensor was prepared by milling 0.04 of NiO/SnO_2_ powder with a binder consisting of an organic solution of terpineol (Sigma Aldrich, St. Louis, MI, USA) and ethyl cellulose (5% in toluene and ethanol solution, TCI, Tokyo, Japan). The milling duration was 30 min, after which the solution was deposited through the use of the spin coating technique (700 rpm for 10 s and 3000 rpm for 30 s) on commercial interdigitated electrodes (IDEs) (Ω Metrohm DropSens, Oviedo, Spain) on a glass substrate. The electrodes were platinum (Pt), and their characteristic distance was 5 μm. After deposition, the sensors were dried at 80 °C for 10 min. Finally, the sensing elements were thermally annealed at 400 °C for 2 h with a 2 °C/min step.

### 2.3. Materials Characterization

The structure of NiO/SnO_2_ was examined via the X-ray Diffraction (XRD) technique using a Bruker AXS D8 Advance copper anode diffractometer (Cu-Kα radiation) equipped with a nickel foil monochromator. It operates at 40 kV and 40 mA over the 2θ/θ collection range of 10°–80° with a scan rate of 0.05°/s. From the XRD pattern, the crystallite size (d) is calculated using Scherer’s Equation (1), as below:

(1) $$d=\frac{0.9\cdot \lambda }{\left(\mathrm{FWHM}\right)\cdot \cos\theta }$$

where λ = 0.154 nm is the wavelength of X-ray radiation, FWHM = Full Width at Half Maximum of the peak corresponding to 2θ angle, and θ is the Bragg angle. The surface morphology was investigated via scanning electron microscopy (SEM) employing a JEOL 7000 microscope (JEOL Ltd., Akishima, Tokyo, Japan) operating at 15 keV, equipped with an energy-dispersive X-ray spectrometer (EDS), which was used for the stoichiometric analysis.

**Figure 1:**
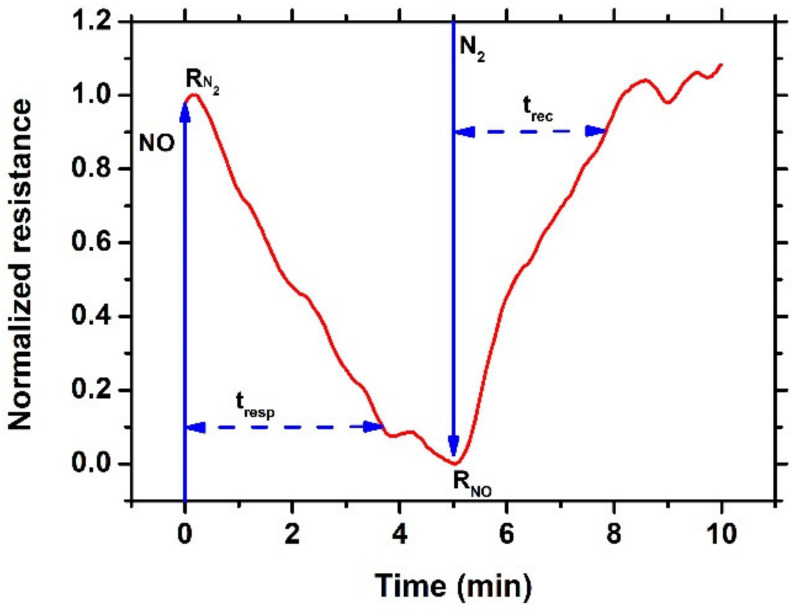
Typical measurement of gas sensing response under 5 min exposure to NO followed by 5 min exposure to N_2_ at room temperature for the NiO/SnO_2_ heterostructure.

The gas sensing performance of the NiO/SnO_2_ heterostructure was studied in a homemade stainless-steel chamber with a volume of 0.7 L. A mechanical pump was used to initially evacuate the chamber as well as to regulate the total pressure in the presence of gases. Two mass flow controllers were used to insert the gases in the chamber with a constant flow of 500 sccm (standard cubic centimeters per minute). Each measurement cycle consisted of a 5 min exposure of the sensor to NO gas, in which the electrical resistance decreased, followed by a 5 min exposure to nitrogen (absence of NO) for the recovery. The NO gas concentrations were 2.5, 5, 7.5 and 10 ppm in nitrogen, which is far below the TLV. The total pressure during the measurements was constant and equal to 800 mbar. In order to monitor the electrical current changes of the sensor upon its interaction with NO gas, a voltage of 1 Volt was applied using a Keithley 6517A electrometer. Τhe procedure was controlled and monitored with a computer using the LabVIEW program. The response R (%) of the sensor is defined by Equation (2)

(2) $$R\left(\%\right)=\frac{R_{N_{2}}-R_{\mathrm{NO}}}{R_{N_{2}}}\cdot 100\%$$

where $R_{N_{2}}$ is the sensor’s resistance value after a 5 min exposure to nitrogen (N_2_) and $R_{\mathrm{NO}}$ is the sensor’s resistance value after a 5 min exposure to NO of different concentrations. Moreover, the response (tresp) and recovery (trec) times are defined as the time that is required for the sensor’s resistance to equal 10% of the $R_{N_{2}}$ in the presence of NO and equal to 90% of the $R_{N_{2}}$ in the presence of N_2_ (absence of NO). In Figure 1, a typical measurement of gas sensing response under a 5 min exposure to NO followed by a 5 min exposure to N_2_ at room temperature for the NiO/SnO_2_ heterostructure, is presented.

It should be noted at this point that the recovery of the sensor has taken place using N_2_ instead of air in order to avoid the formation of NO_2_ as a product of the reaction between NO and O_2_. In addition, it has been reported [41] that there is no difference in the sensors’ behavior if the recovery takes place using N_2_ or air.

Finally, in the case of the synthetic procedure, we synthesized the materials under the same conditions more than 10 times, and we used powders with the same structural morphological and sensing properties. The synthetic procedure is reproducible. Moreover, in all cases, the materials were measured 3 times and showed exactly the same results.

## 3. Results

**Figure 2:**
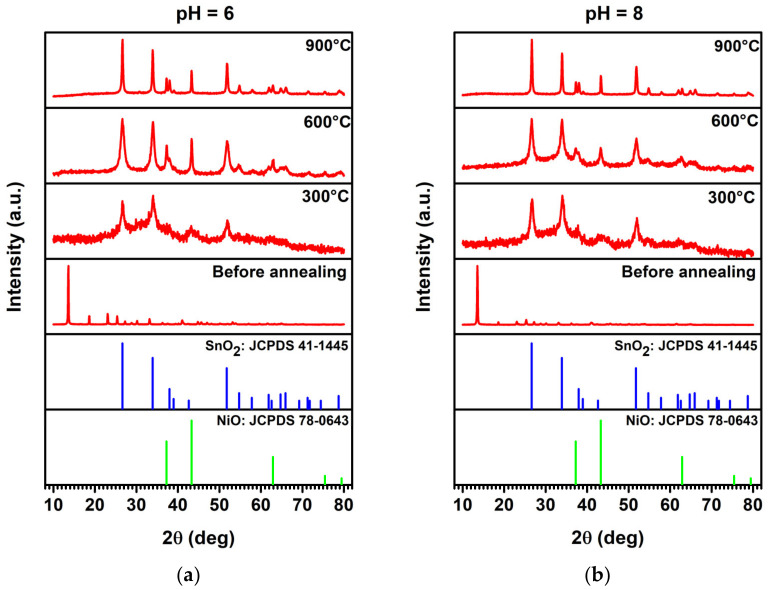
XRD patterns of NiO/SnO_2_ heterostructures prepared using solutions with pH equals (**a**) 6 and (**b**) 8 before and after their thermal annealing.

In Figure 2, the XRD patterns of the NiO/SnO_2_ heterostructures prepared from solutions with pH equal to 6 (a) and 8 (b) before and after their thermal annealing are presented. It can be seen that the as-prepared material was of low crystallinity, while after the thermal annealing, a mix of NiO and SnO_2_ phases with strong peaks was observed, confirming the formation of the heterostructure, which is also in accordance with the literature [32]. The crystalline planes were identified using JCPDS card No. 41-1445 and 78-0643 for SnO_2_ and NiO, respectively. In particular, for pH = 6, at 300 °C, the peaks that are observed at 2θ = 26.62°, 34.02° and 51.86° correspond to the (110), (101) and (211) planes of SnO_2_, respectively, while the one at 2θ = 43.11° corresponds to the (202) plane of NiO. For the heterostructures that were annealed at 600 °C, the peaks at 2θ = 26.63°, 33.98°, 37.98°, 51.86°, and 54.62° correspond to the (110), (101), (111), (211), and (220) planes of SnO_2_, respectively, while those at 2θ = 37.29°, 43.32°, and 62.96° correspond to (202) (021) and (024) planes of NiO, respectively. Finally, for heterostructures that were annealed at 900 °C, the peaks at 26.66°, 33.92°, 38.00°, 51.82°, 54.82°, 57.91°, 61.05°, 64.78°, 66.04° and 69.25° correspond to (110), (101), (111), (211), (220), (002), (310), (112), (301) and (311) planes of SnO_2_, respectively, while those at 2θ = 37.25°, 43.28°, 62.92°, 71.36° and 75.36° correspond to (021), (202), (024), (312) and (223) planes of NiO, respectively. In the same way, for the heterostructures prepared with pH = 8, the observed peaks and the corresponding planes were 2θ = 26.74° (110), 34.02° (101), 52.01° (211) of SnO_2_ and 2θ = 43.09° (202) at the annealing temperature of 300 °C, while for the annealing temperature of 600 °C, the observed peaks and planes were 2θ = 26.60° (110), 33.97° (101), 54.61° (220) και and 62.62° (021) for SnO_2_ and 2θ = 37.34° (021), 43.26° (202) and 62.95° (024) for NiO. Finally, for the annealing temperature of 900 °C, the peaks and the corresponding planes are observed at 2θ = 26.66° (110), 33.95° (101), 38.05° (111), 51.87° (210), 54.81° (220), 57.93° (002), 62.03° (310), 64.86° (112) and 66.08° (301) for SnO_2_ and at 2θ = 43.31° (202), 37.30° (021), 62.87° (024), 71.33° (312) and 75.32° (223), for NiO. A summary of the planes of each heterostructure material is presented in Table 1.

Moreover, in Table 2, the crystallite size of the NiO/SnO_2_ heterostructure, which was calculated for the (110) plane of SnO_2_ using Equation (1), is presented. It can be seen that the crystallite size remains unaffected by the pH of the solution, while it is strongly dependent on the annealing temperature. Specifically, there is a decrease in crystallite size between 300 °C and 600 °C, which can be attributed to the formation of more planes from both materials (Table 1). In contrast, there is a notable increase in the crystallite size at the annealing temperature of 900 °C, which, in combination with the increase in the number of different crystallographic planes formed at this temperature (Table 1), confirms the high crystallinity of the heterostructure.

**Figure 3:**
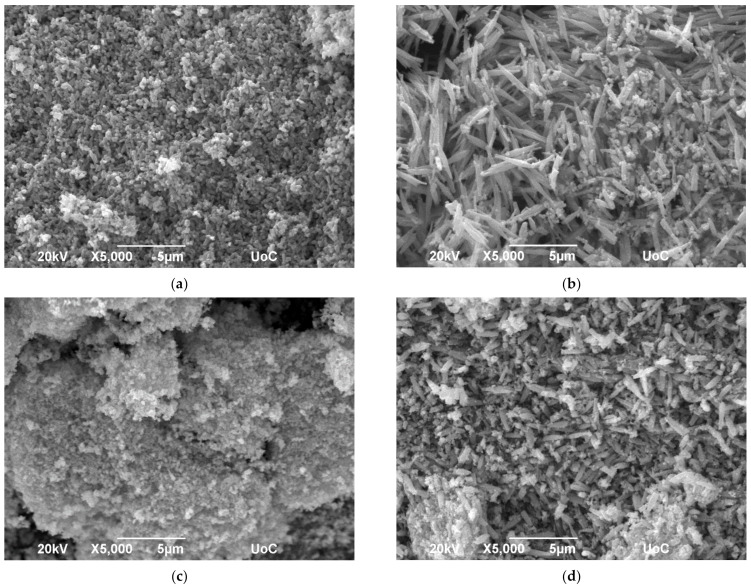

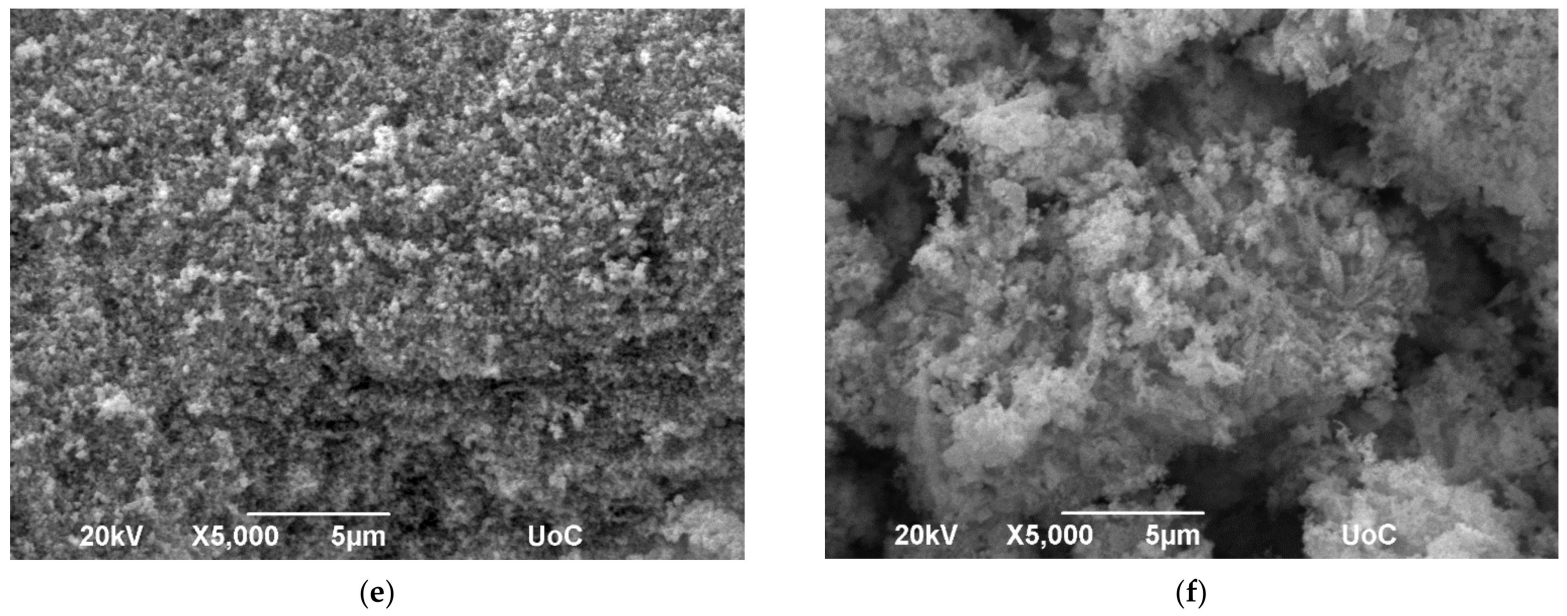
SEM images of NiO/SnO_2_ heterostructures prepared at pH = 6 and annealed at (**a**) 300 °C, (**b**) 600 °C and (**c**) 900 °C as well as at pH = 8 and annealed at (**d**) 300 °C, (**e**) 600 °C and (**f**) 900 °C.

The surface morphology of the heterostructure was investigated via scanning electron microscopy, the images of which are presented in Figure 3a–f. From these, the two different materials, NiO and SnO_2_, can be distinguished, indicating the formation of the heterostructure. The morphology of the heterostructure prepared with pH = 6 and annealed at 300 °C as well as of those prepared with pH = 8 and annealed at 300 °C and 600 °C consist mainly of rods (SnO_2_) with different sizes depending on both the pH and annealing temperature, accompanied with nanosized cubes of NiO. This is in agreement with the stoichiometric analysis presented in Table 2, in which the ratio NiO/SnO_2_ for the above-mentioned heterostructures is almost equal to 35%/65%, thus containing the least amount of NiO. In the other heterostructures, a sponge-like morphology can be observed; however, the two materials can be distinguished, even in the heterostructure in which the ratio NiO/SnO_2_ is almost 44%/56%, which is the highest NiO content.

**Figure 4:**
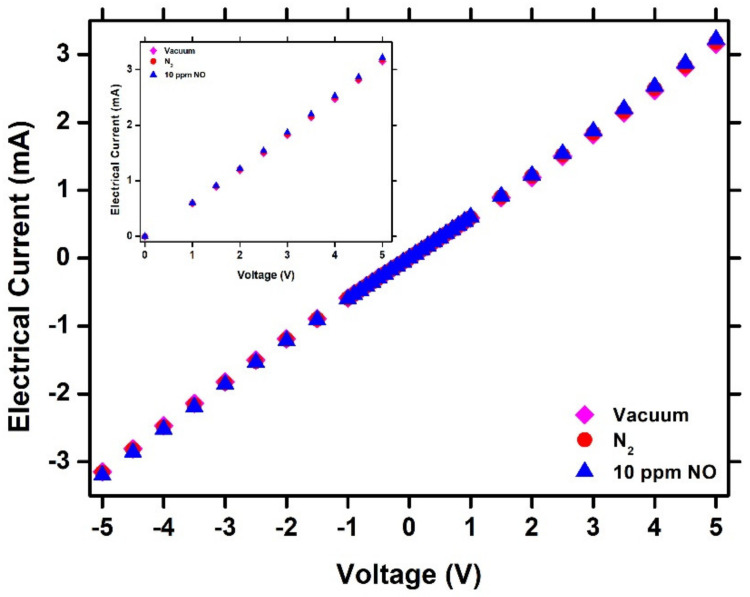
I–V characteristic curve of NiO/SnO_2_ heterostructure under vacuum, N_2_ and 10 ppm NO. In the inset, the part of I–V curve for V = 0–5 V is presented with better analysis.

In order to characterize the electric behavior of the heterostructure, a voltage between −5 V and 5 V was applied, and the electrical current was monitored. In Figure 4, the I–V characteristic curve of the heterostructure under different vacuum atmospheres and nitrogen concentrations at 10 ppm NO is presented. It can be seen that there is a linear relationship between the electrical current and the applied voltage, indicating Ohmic contact between the NiO/SnO_2_ and the Pt metal of the IDEs, independent of the environmental conditions. Moreover, in the inset of Figure 4, the part of the I–V curve, when varying the applied voltage from 0 V to 5 V, is presented in order to differentiate the electrical current values under 10 ppm NO.

**Figure 5:**
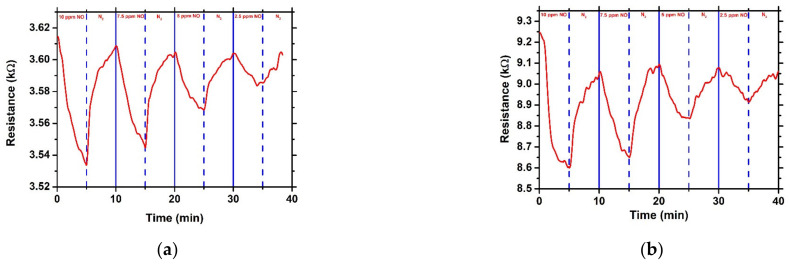
Resistance variation as function of time under exposure to different concentrations of NO for NiO/SnO_2_ heterostructures prepared at pH (**a**) 6 and (**b**) 8 followed by thermal annealing at 900 °C.

**Figure 6:**
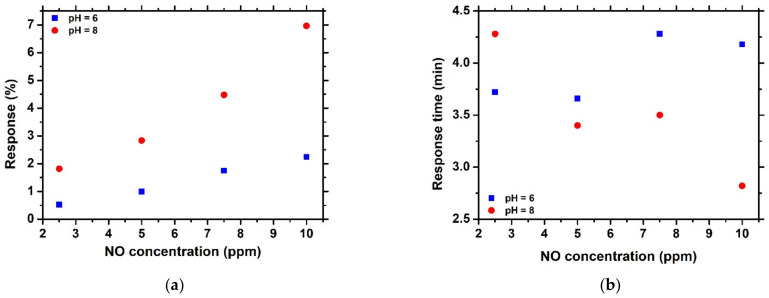
(**a**) Response (%) and (**b**) response time (t_resp_) as a function of NO gas concentration for NiO/SnO_2_ heterostructures that were prepared at different pH.

All NiO/SnO_2_ heterostructures were tested against NO gas at room temperature. In Figure 5a,b, the resistance variation with time of the heterostructures that were prepared at pH = 6 and 8 and annealed at 900 °C is presented. The cycle duration consisted of 5 min exposure to different concentrations of NO, followed by a 5 min exposure to N_2_ for recovery at room temperature. It can be seen that both materials had the ability to detect NO at 2.5 ppm at room temperature. In particular, the sensor prepared with pH = 6 showed a response of 2.2% and 0.5%, while the one prepared with pH = 8 showed a response of 6.9% and 1.8% at 10 ppm and 2.5 ppm, respectively. In addition, in Figure 6a, the response of each heterostructure, as a function of NO concentration, is presented. It is obvious that the heterostructure that was prepared at pH = 8 shows a greater response than that with pH = 6. Moreover, the response of the former increased at a higher rate than the latter, indicating that the pH affects the response to NO gas, probably due to higher porosity (Figure 3f) that appeared to have the heterostructure that was prepared at pH = 8. The structural parameters, such as crystallite size, play a major role in gas sensing performance. However, in our case, crystallite sizes for the heterostructures prepared with different pH are similar and, as a result, the enhanced response in terms of NO can be attributed to the higher NiO content in the heterostructure or to the different morphology.

The response time of the heterostructures as a function of NO concentration is presented in Figure 6a,b. The heterostructure prepared at pH = 6 showed an almost constant response time of about 4 min, indicating that the amount of NO that is adsorbed on the surface is the same, independent of NO concentration, which is probably attributed to low porosity. In contrast, the higher porosity of the other heterostructure (pH = 8) resulted in decreased response time with concentration due to the fact that a greater amount of NO can be adsorbed on the surface. Porosity plays an important role in the gas sensing performance since the higher it is, the greater the gas content that will interact with the sensing element. As a result, the response will be increased, while the response time will be decreased. As far as the recovery time is concerned, it was found to have a mean value of about 4 min for both heterostructures.

The sensing mechanism that governs the interaction between NO gas and the heterostructure can be summarized in Equations (3)–(7) [11,42]

(3) $$NO_{g}+e^{-}↔NO_{ads}^{-}$$

(4) $$2NO_{ads}^{-}\to N_{2}O_{ads}^{2-}$$

(5) $$N_{2}O_{2}^{2-}{}_{ads}\to N_{2}O_{ads}+O_{ads}^{-}$$

(6) $$N_{2}O_{ads}^{-}\to N_{2}O_{g}+e^{-}$$

(7) $$O_{ads}^{-}+NO_{g}\to N_{2}O_{g}+e^{-}$$

which are based on adsorption and desorption of NO gas on the surface of the heterostructure.

**Figure 7:**
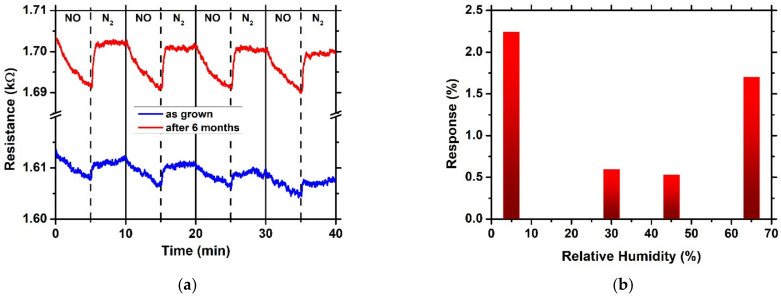
(**a**) Repeatability and stability and (**b**) response as a function of relative humidity of the NiO/SnO_2_ sensor toward 10 ppm NO at room temperature.

In order to examine the repeatability as well as the stability of the sensor, it was tested against 10 ppm NO for four cycles of 5 min NO — 5 min N_2_, both the as-prepared sensor and after it had been stored in an ambient environment for 6 months. In Figure 7a, it can be seen that the sensor showed excellent repeatability as well as stability. Moreover, the enhanced response of the sensor after 6 months of storage can be observed, probably due to the O_2_ adsorption on the surface of the heterostructure.

Additionally, the sensor prepared at pH = 6 was evaluated under different values of relative humidity, RH, as presented in Figure 7b. The response decreased as the RH increased from 5% to 30% and 45%; however, it increased when the RH was equal to 65%. The decrease in response in relation to relative humidity can be related to the fact that water molecules decrease the active sites in which the NO are adsorbed [7].

In Table 3, the gas-sensing characteristics of materials that have been used as NO-sensing elements are summarized. It can be seen that only a few of them operated at room temperature. From this, it can be concluded that the NiO/SnO_2_ heterostructure can be a possible candidate sensing element for NO detection at room temperature.

Finally, it should be noted that NiO/SnO_2_ heterostructures were also tested against other gases, such as ozone, hydrogen and carbon dioxide, showing no response, indicating a selective response to NO gas. However, more experiments must be performed in the future.

## 4. Conclusions

In the present work, the NiO/SnO_2_ heterostructure was examined for the first time as a NO gas sensor at room temperature, showing encouraging results. In particular, the heterostructure was grown using a polyol process, varying the pH of the solution, and the received powder was further annealed at 300 °C, 600 °C and 900 °C. The heterostructures were characterized using the X-ray diffraction technique, confirming the two different phases of SnO_2_ and NiO, while their ratio was determined via energy-dispersive spectroscopy. In addition, the surface morphology was studied using scanning electron microscopy, revealing that the formed morphologies are dependent mainly on the annealing temperature and less on the pH. The heterostructures were tested toward NO gas at room temperature, showing a response of 1.8% at 2.5 ppm, far below the TLV, and a response time of 4.3 min. Compared to other NO gas sensors operating at room temperature, it can be concluded that NiO/SnO_2_ is a candidate sensing material for NO detection in areas that are burdened by NO emissions caused by automobiles, factories, etc. Thus, by controlling the NO concentrations, diseases such as asthma can be avoided.

## Figures and Tables

**Table 1:** Crystallographic planes of the NiO/SnO_2_ heterostructure.

pH	Annealing Temperature (°C)	Material	Planes
**6**	300	NiO	(200)
		SnO_2_	(110), (101)
	600	NiO	(202), (021), (024)
		SnO_2_	(110), (101), (111), (211), (220)
	900	NiO	(202), (021), (024), (312), (223)
		SnO_2_	(110), (101), (111), (210), (220), (002), (310), (112), (301)
**8**	300	NiO	(202)
		SnO_2_	(110), (101), (211)
	600	NiO	(202), (021), (024)
		SnO_2_	(110), (101), (220), (021)
	900	NiO	(202), (021), (024), (312), (223)
		SnO_2_	(110), (101), (111), (210), (220), (002), (310), (112), (301)

**Table 2:** Stoichiometric analysis and crystallite size (calculated at (110) plane of SnO_2_) of the NiO/SnO_2_ heterostructure.

pH	Annealing Temperature (°C)	NiO/SnO_2_ (%)/(%)	Crystallite Size (nm)
**6**	300	35.66/64.34	14.0
	600	40.85/59.15	9.4
	**900**	**35.71/64.29**	30.1
**8**	300	35.31/64.69	14.1
	600	36.10/63.90	9.9
	**900**	**43.64/56.36**	33.3

**Table 3:** Summarize of NO gas sensing elements.

Material	Operation Temperature (°C)	Response	Concentration (ppm)	Response Time (s)	Ref.
**ZnO:ZnGa_2_O_4_**	400	28.6 *	100	8	[1]
**CuO NPs**	50	3 **	100	<2.5	[2]
**Ag:ZnO**	100	53.28% ***	0.0216	-	[6]
**SnO_2_**	160	33.3 *	0.5	214	[7]
**N:rGO**	RT	1.7 *	1	-	[18]
**WO_3_**	150	3.22 *	0.1	-	[11]
**Pd loaded Co_3_O_4_**	200	1.16 *	0.2	456	[12]
**PCDTBT** **Conductive polymer**	RT	80.6% ***	100	300	[19]
**TiO_2_-rGO**	RT	7.1 *	2.75	440	[20]
**Tb_2_O_3_/ZnO**	180	28.3 *	1	208	[13]
**NiO/SnO_2_ (pH = 6)**	RT	0.5 *	2.5	223	This work
**NiO/SnO_2_ (pH = 8)**	RT	1.8 *	2.5	257	This work

* R_g_/R_a_, ** R_a_/R_g_,*** [(R_g_ − R_a_)/R_a_] × 100%.

## Data Availability

The data presented in this study are available on request from the corresponding author. The data are not publicly available due to privacy.

## References

[1] Singh A.K., Yen C.C., Wen C.F., Horng R.H., Wuu D.S. (2023). Growth and Characterization of Sputtered ZnO:ZnGa_2_O_4_ Dual-Phase Films on Sapphire Substrates for NO Gas-Sensing Applications. ACS Appl. Electron. Mater..

[2] Censabella M., Iacono V., Scandurra A., Moulaee K., Neri G., Ruffino F., Mirabella S. (2022). Low Temperature Detection of Nitric Oxide by CuO Nanoparticles Synthesized by Pulsed Laser Ablation. Sens. Actuators B Chem..

[3] Li W., Geng X., Guo Y., Rong J., Gong Y., Wu L., Zhang X., Li P., Xu J., Cheng G. (2011). Reduced Graphene Oxide Electrically Contacted Graphene Sensor for Highly Sensitive Nitric Oxide Detection. ACS Nano.

[4] Lim N., Kim K.H., Byun Y.T. (2021). Preparation of Defected SWCNTs Decorated with En-APTAS for Application in High-Performance Nitric Oxide Gas Detection. Nanoscale.

[5] Chung M.H., Hwang R.C., Chiu J.J., Yang M.W., Hung T.T., Shen C.Y. (2019). Enhanced Sensitive Surface Acoustic Wave Device Designed for Nitric Oxide Gas Detection. Sens. Mater..

[6] Tsai Y.T., Chang S.J., Ji L.W., Hsiao Y.J., Tang I.T., Lu H.Y., Chu Y.L. (2018). High Sensitivity of NO Gas Sensors Based on Novel Ag-Doped ZnO Nanoflowers Enhanced with a UV Light-Emitting Diode. ACS Omega.

[7] Su P., Li W., Zhang J., Xie X. (2022). Chemiresistive Gas Sensor Based on Electrospun Hollow SnO_2_ Nanotubes for Detecting NO at the Ppb Level. Vacuum.

[8] Rispandi, Putro D.T., Chu C.S. Optical Dual Sensor Single Fiber Pt(II) Complex Coated Perovskite Quantum Dots Green for Oxygen and Nitric Oxide Gas Detection. Proceedings of the 2022 IET International Conference on Engineering Technologies and Applications (IET-ICETA).

[9] Wang S.H., Kuo S.H., Shen C.Y. (2011). A Nitric Oxide Gas Sensor Based on Rayleigh Surface Acoustic Wave Resonator for Room Temperature Operation. Sens. Actuators B Chem..

[10] Wang S.H., Shen C.Y., Huang H.M., Shih Y.C. (2014). Rayleigh Surface Acoustic Wave Sensor for Ppb-Level Nitric Oxide Gas Sensing. Sens. Actuators A Phys..

[11] Lin C.H., Chang S.J., Hsueh T.J. (2017). A WO3 Nanoparticles NO Gas Sensor Prepared by Hot-Wire CVD. IEEE Electron Device Lett..

[12] Akamatsu T., Itoh T., Izu N., Shin W., Sato K. (2015). Sensing Properties of Pd-Loaded CO_3_O_4_ Film for a Ppb-Level NO Gas Sensor. Sensors.

[13] Wei T., Li W., Zhang J., Xie X. (2023). Synthesis of Tb_2_O_3_/ZnO Composite Nanofibers via Electrospinning as Chemiresistive Gas Sensor for Detecting NO Gas. J. Alloys Compd..

[14] Soltabayev B., Ajjaq A., Yergaliuly G., Kadyrov Y., Turlybekuly A., Acar S., Mentbayeva A. (2023). Ultrasensitive Nitric Oxide Gas Sensors Based on Ti-Doped ZnO Nanofilms Prepared by RF Magnetron Sputtering System. J. Alloys Compd..

[15] Madhaiyan G., Tung T.W., Zan H.W., Meng H.F., Lu C.J., Ansari A., Chuang W.T., Lin H.C. (2020). UV-Enhanced Room-Temperature Ultrasensitive NO Gas Sensor with Vertical Channel Nano-Porous Organic Diodes. Sens. Actuators B Chem..

[16] Karaduman Er I., Çağirtekin A.O., Çorlu T., Yildirim M.A., Ateş A., Acar S. (2019). Low-Level NO Gas Sensing Properties of Zn1-XSnxO Nanostructure Sensors under UV Light Irradiation at Room Temperature. Bull. Mater. Sci..

[17] Li M., Zou C., Liang F., Hou E., Huang H., Lin J. (2023). Preparation and Gas-Sensing Performance under Photoactivation of TiO_2_ Nanotubes/Carbon Nanotubes/ZnS Quantum Dots Gas-Sensitive Materials. Mater. Today Commun..

[18] Chang Y.S., Chen F.K., Tsai D.C., Kuo B.H., Shieu F.S. (2021). N-Doped Reduced Graphene Oxide for Room-Temperature NO Gas Sensors. Sci. Rep..

[19] Gusain A., Joshi N.J., Varde P.V., Aswal D.K. (2017). Flexible NO Gas Sensor Based on Conducting Polymer Poly[N-9′-Heptadecanyl-2,7-Carbazole-Alt-5,5-(4′,7′-Di-2-Thienyl-2′,1′,3′-Benzothiadiazole)] (PCDTBT). Sens. Actuators B Chem..

[20] Kuchi C., Naresh B., Reddy P.S. (2021). In Situ TiO_2_-RGO Nanocomposite for Low Concentration NO Gas Sensor. ECS J. Solid State Sci. Technol..

[21] Barsan N., Schweizer-Berberich M., Göpel W. (1999). Fundamental and Practical Aspects in the Design of Nanoscaled SnO_2_ Gas Sensors: A Status Report. Fresenius J. Anal. Chem..

[22] Das S., Jayaraman V. (2014). SnO_2_: A Comprehensive Review on Structures and Gas Sensors. Prog. Mater. Sci..

[23] Yuliarto B., Gumilar G., Septiani N.L.W. (2015). SnO_2_ Nanostructure as Pollutant Gas Sensors: Synthesis, Sensing Performances, and Mechanism. Adv. Mater. Sci. Eng..

[24] Masuda Y. (2022). Recent Advances in SnO_2_ Nanostructure Based Gas Sensors. Sens. Actuators B Chem..

[25] Kumar R., Mamta, Kumari R., Singh V.N. (2023). SnO_2_-Based NO_2_ Gas Sensor with Outstanding Sensing Performance at Room Temperature. Micromachines.

[26] Tyagi P., Sharma A., Tomar M., Gupta V. (2016). Metal Oxide Catalyst Assisted SnO_2_ Thin Film Based SO_2_ Gas Sensor. Sens. Actuators B Chem..

[27] Lu S., Zhang Y., Liu J., Li H.Y., Hu Z., Luo X., Gao N., Zhang B., Jiang J., Zhong A. (2021). Sensitive H2 Gas Sensors Based on SnO_2_ Nanowires. Sens. Actuators B Chem..

[28] Bunpang K., Wisitsoraat A., Tuantranont A., Singkammo S., Phanichphant S., Liewhiran C. (2019). Highly Selective and Sensitive CH_4_ Gas Sensors Based on Flame-Spray-Made Cr-Doped SnO_2_ Particulate Films. Sens. Actuators B Chem..

[29] Ayesh A.I., Alyafei A.A., Anjum R.S., Mohamed R.M., Abuharb M.B., Salah B., El-Muraikhi M. (2019). Production of Sensitive Gas Sensors Using CuO/SnO_2_ Nanoparticles. Appl. Phys. A Mater. Sci. Process..

[30] Wang L., Lou Z., Zhang R., Zhou T., Deng J., Zhang T. (2016). Hybrid CO_3_O_4_/SnO_2_ Core-Shell Nanospheres as Real-Time Rapid-Response Sensors for Ammonia Gas. ACS Appl. Mater. Interfaces.

[31] Tyagi P., Sharma A., Tomar M., Gupta V. (2016). Low Temperature Operated NiO-SnO_2_ Heterostructured SO_2_ Gas Sensor. AIP Conf. Proc..

[32] Meng D., Liu D., Wang G., Shen Y., San X., Li M., Meng F. (2018). Low-Temperature Formaldehyde Gas Sensors Based on NiO-SnO_2_ Heterojunction Microflowers Assembled by Thin Porous Nanosheets. Sens. Actuators B Chem..

[33] Wang X., Wang S., Tian J., Cui H., Wang X. (2022). Synthesis of 1D SnO_2_ Nanorods/2D NiO Porous Nanosheets p-n Heterostructures for Enhanced Ethanol Gas Sensing Performance. Vacuum.

[34] Liang Y., Li H., Zhao X., Xue L., Tang L., Xue F., Yu T., Yang Y. (2023). Crystal Facets-Controlled NiO/SnO_2_ p-n Heterostructures with Engineered Surface and Interface towards Triethylamine Sensing. J. Alloys Compd..

[35] Ma Q., Li H., Guo J., Chu S., Zhang Q., Lin Z. (2021). Available Surface Electronic Transmission of Porous SnO_2_/NiO Hollow Nanofibers for the Enhanced Gas-Sensing Performance toward n-Butanol. Mater. Sci. Semicond. Process..

[36] Majder-Łopatka M., Wesierski T., Dmochowska A., Salamonowicz Z., Polańczyk A. (2020). The Influence of Hydrogen on the Indications of the Electrochemical Carbon Monoxide Sensors. Sustainability.

[37] Ye W., Jiang C., Lei J., Feng Z., Xiong D., He M. (2023). Fluorine-Doped and Carbon-Coated Porous SnO_2_-NiO Composite as a High Performance Anode Material for Lithium Ion Batteries. Chem. Phys. Lett..

[38] Muruganandam S., Kannan S., Anishia S.R., Krishnan P. (2023). Electrochemical Performance of Yttrium Doped SnO_2_–NiO Nanocomposite for Energy Storage Applications. J. Phys. Chem. Solids.

[39] Maarisetty D., Baral S.S. (2021). Effect of Defects on Optical, Electronic, and Interface Properties of NiO/SnO_2_ Heterostructures: Dual-Functional Solar Photocatalytic H_2_ Production and RhB Degradation. ACS Appl. Mater. Interfaces.

[40] Athira M., Shafna K.K.F., Angappane S. (2023). Enhanced Photodetector Performance of SnO_2_/NiO Heterojunction via Au Incorporation. Semicond. Sci. Technol..

[41] Jeon J.Y., Kang B.C., Byun Y.T., Ha T.J. (2019). High-Performance Gas Sensors Based on Single-Wall Carbon Nanotube Random Networks for the Detection of Nitric Oxide down to the Ppb-Level. Nanoscale.

[42] (2014). Bei-Yu Chang, Chen-Yang Wang, Hsiao-Fang Lai, Ren-Jang Wua, Murthy Chavali Evaluation of Pt/In2O3–WO3 Nano Powder Ultra-Trace Level NO Gas Sensor. J. Taiwan Inst. Chem. Eng..

